# Children and Creeds

**Published:** 1889-08-31

**Authors:** 


The Hospital

A Weekly Institutional Journal of

Science, Medicine, Nursing, and Philanthropy.

*For Hospitals, Asylums, Medical Practitioners, Students, Nurses, Families, Parishes, Congregations, and the Charitable Public.*

Vol. VI. No. 153.] [August 31, 1889.

### CHILDREN AND CREEDS.

Practical persons, who understand and value the practical aspects of Christianity, Judaism, and all other moral faiths, regard with pleasure any new departure which tends to unite in heart, if not in opinion, all well-conditioned and honest-intentioned men and women. No man who has ever had occasion to blush inwardly at the frequent discovery of his own narrowness of intellect and of sympathy, will speak very harshly of those who hold firmly by a creed which they honestly believe. Convictions, in this age at any rate, are often difficult to come by, and when they are obtained there is a very natural reluctance to let them go. We confess our confidence in those who have convictions, and who stand by them. At the same time we cannot help feeling that there ought to be, somewhere in Nature or in mind, a platform on which all honest men might conscientiously stand side by side, and feel nothing but brotherly kindness towards one another. If it be true that there is "One God and Father of us all," there must be, somewhere or other, a place or places in which men of honest intent can feel at home in one another's company. There are such places, no doubt, and every man who is either reasonable or well-intentioned, or both, will do his utmost to multiply them. He who, on the other hand, regards such places with suspicion, and speaks or acts against them, is either deficient in intellect or unworthy in heart.

The Children's Country Holidays Fund—which we have often pleaded for in these columns, but which we are not at this moment so desirous of enriching in material resources as of holding up as a platform of unity—is a society well worthy of the consideration of men of practical goodness belonging to every creed and party. In a brief "script" now before us the society urges, as one of its chief claims to consideration, the fact that it is "unsectarian." It is true, says the "script," that the clergy of the English Church are amongst the most active supporters of the society, and that the names of several bishops appear on its list of vice-presidents. But, by way of set-off, there are also amongst the vice-presidents such staunch representatives of Nonconformity as the Rev. Dr. Allon and Mr. Mundella. The name of Cardinal Manning is also to be found among them, as well as the names of other persons in whom the public are accustomed to place confidence. The council makes every possible effort to ensure that no child shall lose his country holiday on account of his creed.

What it is desired to point out and emphasise is, that the society is in no sense a Church of England society, although it is largely supported by members of the Church of England. It is an English society, and tries to select as its beneficiaries those who most need the kind of aid it can render—that is, a more or less prolonged visit to the country. The selected cases are the children of parents who profess every kind of religion, and some of them no religion at all. One might suppose that it would be a matter of no great moment even if the children all stayed with Church families, and went to church on Sundays. Few people can be found who will avow that a Churchman must necessarily be a maker of proselytes, or that the service of the Church of England can in any way injure the mind or the morals of a child. But, as a matter of fact, the society spares no pains to find homes for the children in which the faith of their parents is professed. Boys and girls whose parents belong to the Church of England are boarded with cottagers who attend that Church, and are taken to the ordinary services. Children whose parents are Congregationalists, Wesleyans, Baptists, or Nonconformists of any other kind are as far as possible placed with families belonging to these denominations, and are expected to go to chapel with them. There is no difficulty in finding suitable homes in country villages for those who belong to the larger Nonconforming Churches. In the case of the children of Jews and Catholics, however, very considerable difficulty is sometimes experienced. But, thanks to the willingness of the parents to yield reasonable concessions, and to the energy of the society in making all possible efforts to meet all reasonable needs, it is found that both Catholics and Jews can be accommodated at a certain number of places without any strain being put upon the consciences of scrupulous parents. This determination on the part of the society is extremely praiseworthy, and will be very fully and warmly approved by all people who have sufficient tenderness of conscience to make them regard with sympathy the tender consciences of others. It has been arranged, by special subscriptions among Jews themselves, to supply Jewish food from certain appointed centres; and thus Jew and Christian, Protestant and Catholic, can meet, and talk, and play together to their mutual pleasure and advantage.

The council of the fund, although they speak with great mildness, think it is a pity that the leading Nonconformists of London do not co-operate more actively with them. It is to be presumed, therefore, that Nonconformists do not support the society to any very large extent. What can be the reason of that? On the surface there is no evident reason. Is there anything out of sight, any real or fancied grievance which shuts up Nonconformist sympathy? There are a number of Nonconformists who still hold themselves aggrieved by what they consider the sectarianism of London hospitals. There is, however, no practical sectarianism of any moment connected with the hospitals. We have every reason to believe that the same may be said, and with even more emphasis, of the Children's Country Holidays Fund. It surely is not for Nonconformists to exaggerate small occasions of difference. No doubt a Nonconformist may fairly enough ask why he should in any circumstances be placed at a disadvantage in comparison with a Churchman? There is no reason why; and this society avoids with the utmost carefulness "even the very appearance of evil." This is a practical world, and Nonconformists should remember that for many years they were a minority of their fellow-countrymen, and minorities are apt at all times to receive less consideration than majorities. In every case, therefore, where no practical grievance can be alleged, they should hold it their duty and see it to be a truly patriotic policy to unite as fully and heartily as possible with all classes of their fellow-countrymen.

It is neither possible nor desirable to enforce uniformity of opinion or of ecclesiastical practice. In religion and politics men will always differ, and it is well they should. Intellectual conflict is the birth pang of many a new truth, and the grinding and refurbishing of many an old one. But conflict does not always mean hatred, and should never mean it among men who speak the same tongue and front the world under the same flag. In the presence of the dead strife is hushed, and the dogs of passion are compelled to lie down. If the face of the dead be sufficient to silence the raging tongue and to still the passionate heart, how much more the unknowing, uncomprehending face of the innocent and wondering child! What recks he of definitions and of formulas, of dialectics and of anathemas? A little child was once placed in the midst of contenders by the great Master as an example of ingenuous simplicity which was declared to be of more worth than all their contentions. His modern representative may well, at this age, be placed in the midst of modern contenders as a symbol and centre of practical unity and peace. The child of the Children's Country Holidays Fund is enfeebled by civilisation, and pale with the hunger of pure air and Nature's sights and sounds. To him, as to God's own symbol of simplicity and friendliness, let all men come; for his sake let them be at peace with one another; and to him let them do good for the sake of goodness alone, and out of a generous heart willingly.

